# Altered thalamic neurotransmitters metabolism and functional connectivity during the development of chronic constriction injury induced neuropathic pain

**DOI:** 10.1186/s40659-020-00303-5

**Published:** 2020-08-26

**Authors:** Zhifu Wang, Sheng Huang, Xiangmei Yu, Long Li, Minguang Yang, Shengxiang Liang, Weilin Liu, Jing Tao

**Affiliations:** 1 College of Integrated Traditional Chinese and Western Medicine, Fujian University of Traditional Chinese Medicine, Fuzhou, 350122 China; 2 National-Local Joint Engineering Research Center of Rehabilitation Medicine Technology, Fujian University of Traditional Chinese Medicine, Fuzhou, 350122 Fujian China; 3 Fujian Collaborative Innovation Center for Rehabilitation Technology, Fuzhou, 350122 Fujian China; 4 College of Rehabilitation Medicine, Fujian University of Traditional Chinese Medicine, Fuzhou, 350122 China

**Keywords:** Chronic pain, N-acetylaspartate (NAA), Glutamate (Glu), Central sensitization, Thalamic functional connectivity

## Abstract

**Background:**

To investigate the thalamic neurotransmitters and functional connections in the development of chronic constriction injury (CCI)-induced neuropathic pain.

**Methods:**

The paw withdrawal threshold was measured by mechanical stimulation the right hind paw with the von frey hair in the rats of CCI-induced neuropathic pain. The N-acetylaspartate (NAA) and Glutamate (Glu) in thalamus were detected by magnetic resonance spectrum (MRS) process. The thalamic functional connectivity with other brain regions was scanned by functional magnetic resonance image (fMRI).

**Results:**

The paw withdrawal threshold of the ipsilateral side showed a noticeable decline during the pathological process. Increased concentrations of Glu and decreased levels of NAA in the thalamus were significantly correlated with mechanical allodynia in the neuropathic pain states. The thalamic regional homogeneity (ReHo) decreased during the process of neuropathic pain. The functional connectivity among the thalamus with the insula and somatosensory cortex were significantly increased at different time points (7, 14, 21 days) after CCI surgery.

**Conclusion:**

Our study suggests that dynamic changes in thalamic NAA and Glu levels contribute to the thalamic functional connection hyper-excitation during CCI-induced neuropathic pain. Enhanced thalamus-insula functional connection might have a significant effect on the occurrence of neuropathic pain.

## Introduction

Neuropathic pain due to injuries or lesions in the peripheral nervous system is always accompanied by allodynia, hyperalgesia, and even numbness [1, 2]. The thalamus, which is the significant area activated as a response to noxious stimulation in normal subjects, has remained the focus of attention in the field of pain research in the past century [3]. Indeed, the thalamus becomes sensitized after a pain attack, reducing the hyperalgesia and allodynia threshold. Clinical studies have shown increased neural activity when patients encounter peripheral nerve injury [4, 5]. The reduction in the thalamic neural response threshold has also been investigated in rodent models [6, 7].

Previous studies have revealed that aberrant activity and neurotransmitter alterations in the central nervous system are the significant pathological mechanism in a variety of pain models [8–10]. Thalamic N-acetylaspartate (NAA) has long been recognized as a pathological marker of chronic pain. A variety of in vivo studies have demonstrated that thalamic NAA alteration is critical in the development of chronic pain. Additionally, glutamate (Glu) levels have been reported to distinguishing healthy individuals from patients [11, 12]. Due to their magnetic sensitivity and mass enrichment in vivo, proton nuclei are widely applied in magnetic resonance spectroscopy studies. Abundant ^1^H MRS voxels depict a spectrogram of the concentration of neurometabolic substances in a specific region of the brain [13]. Besides, blood-oxygenation-level dependent (BOLD)-fMRI was used to investigate the intrinsic functional connectivity between the thalamus and other brain regions.

As mentioned above, few studies on whole brain functional activities and neurometabolic changes have provided us with the pathological mechanism of pain in the central nervous system. The response of thalamic alterations to pathological progress has rarely been studied. The purpose of the current study was to further identify thalamic neurotransmitters and functional connectivity alterations in the rat model of CCI-induced neuropathic pain.

## Materials and methods

### Animals

Nineteen adult male SD rats (250 ± 20 g) were divided into two groups (CCI rats, n = 10; Sham rats, n = 9, rats licence No SYXK2019-0007). Rats were housed in plastic cages that were kept in a room with a constant moderate temperature and humidity of 23 ± 2 °C and 55 ± 5%, respectively, without restriction on food and water intake. The light/dark cycle alternated every 12 h. The experiments were performed in strict conformity with the study protocols and ethical guides of Fujian University of Traditional Chinese Medicine (No. FJTCM 2019-006).

### Surgical procedures

Briefly, 2% inhaled isoflurane via a precision vaporizer was used for animal anaesthesia maintenance. A 0.5 cm incision was made in the middle of the right thigh of rats. The sciatic nerve was fully exposed after bluntly separating the layers of tissue, and then was ligated with four 4-0 surgical catgut to maintain slight compression. Muscle and subcutaneous tissues were sutured without no blood exudation in the operative field. Rats were allowed to recover until they regained consciousness and were able to breathe spontaneously in the cages. The surgical procedure in sham-treated rats is consistent with the above mentioned except the ligation of the sciatic nerve.

### Von Frey’s test

The Von-Frey method was adopted for detecting the paw withdrawal latency by the different strengths stimulation to the central part of the paw of rats. The specific operation is as follows: The animals were placed in the test box (10 × 20 × 20 cm) to adapt to the environment and reduce stress. During the test, the animals were put into the experimental environment 15–30 min in advance to adapt to the environment. When the animal is quiet or relaxed, begin the test. In the experiment, 0.6-26 g Von Frey hairs (The Aesthesio® set of 20 monofilaments, USA) were used to vertically stimulate the central part of hind limbs of animals. One stimulation time was 3 s for 5 consecutive times. When Von-Frey hairs are stimulated, the animals produce a positive response accompanied by paw withdraw. The hair stimulus intensity is considered as the sufficient intensity if more than three times of five stimulations appear paw response. On the contrary, it is a negative intensity. When using the sufficient hair gram to induce a positive reaction, change the lower gram for stimulation and record the last smallest positive response gram as the paw withdrawal threshold (PWT). On the other hand, when the hair intensity was ineffective, select the higher hair stimulation until the emergence of positive response. Then, the minimum stimulus intensity was chosen as the paw withdrawal threshold on the affected side of the limb.

### fMRI scanning

After the pain threshold test, the rats were fixed in the prone position with the coils on the scanning platform. During the fMRI scanning process, the anaesthesia isoflurane concentration was kept between 0.5 and 1.0%, while the temperature of the scanning bed was kept near 35 °C. The respiratory and heart rates were monitored throughout the scanning process. The imaging scanning process was conducted using a 7.0 T animal magnetic resonance imaging scanner (Bruker, Germany).

The location sequence was applied first to determine the rat head position and confirm the centre of the imaging field. The relaxation enhancement T2-weighted sequence was executed with the following parameters: TR = 4200 ms, TE = 35 ms, slice thickness = 1 mm, slices = 21, and matrix = 256 × 256. After the end of this sequence scan, oxygen continued to be supplied, and isoflurane was briefly turned off. After the rat's respiration rate was restored to 90 breaths/min, isoflurane was maintained at approximately 0.5% to maintain this respiration rate in the rat. The gradient echoes planar imaging sequence was obtained for the functional MRI data with the following parameters: TR = 2000 ms, TE = 28 ms, slice thickness = 1 mm, slices = 21, and matrix = 64 × 64. SPM software was used to pre-process all functional data, such as time-division rearrangement, head dynamic correction, spatial standardization and smoothing (0.2, 0.2, 0.2 mm of the Gauss nucleus). Because excessive motion cannot be rectified, one CCI rat was excluded in this process. For analysis, the extracerebral area was removed, and only the brain tissue area was preserved. After spatial standardization, the spatial image was filtered and de-linearized, and the thalamus was selected as the region of interest (ROI). The correlation metric among the seed area and other brain regions was calculated, and a functional connectivity map of the rat brain was obtained. To evaluate the trend in the functional links of two groups, three-time points of two groups were taken as independent variables. Individual regional homogeneity (ReHo) maps were generated by calculating Kendall's coefficient of concordance of the time series of a given voxel with those of its nearest neighbour (26 voxels) according to our previous research methods [14]. Functional connectivity was evaluated using seed-based correlational analysis. The time courses of all voxels within the left thalamus were averaged to use as reference time courses.

### ^1^H-MRS spectral processing

At the end of the EPI sequence scanning process, the thalamus was selected as the ROI (3 × 3 × 3 mm^3^) for ^1^H-MRS scanning. Short-TE point-resolved spectroscopy with a water suppression pulse was used for MRS data acquisition: TR = 1500 ms, TE = 20 ms, and number of averages = 256. The post-processing of images and related data were performed by using the workstation TOPSPIN (V3.1, Bruker Biospin, Germany) of the MRI instrument. The peak-area ratio indicates various metabolic concentrations. Creatine (Cr) is used as an internal reference, and the relative quantification of NAA/Cr, Glu/Cr in the brain were recorded as the relative quantification of the substance in the brain. The spectrum peak positions were kept as follow: Cr approximately 3.0 ppm, NAA approximately 2.02 ppm, Glu approximately 2.7 ppm.

Both the fMRI scanning and behavioural tests were performed at different time points of 0, 7, 14, and 21 days after CCI surgery. The differences of PWT, ReHo, spectrum and function connectivity data between the two groups were analyzed by the Analysis Of Variance (ANOVA) and post hoc Tukey test in the SPSS 21.0 software (SPSS, Armonk, NY, USA). The Pearson correlation coefficient was used to analyze the relationship between the spectrum neurotransmitters and behaviour. A *P* value of < 0.05 was considered statistically significant.

## Results

### Changes in mechanical threshold on the days 0, 7, 14, 21 after CCI surgery

**Fig. 1:**
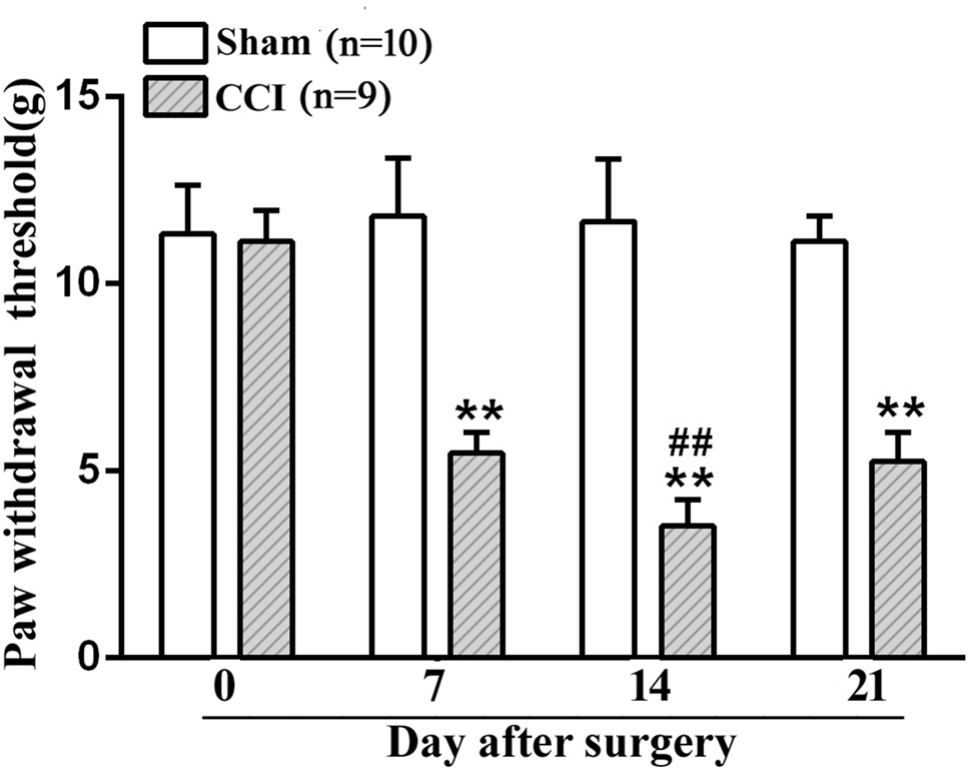
Mechanical pain behavior in the CCI-induced neuropathic pain

As illustrated in Fig. 1, compared to the sham group, the paw withdrawal threshold of the ipsilateral side showed a prominent decline in rats on day 7 (5.48 ± 0.56 g vs 11.80 ± 1.57 g, **P < 0.01), day 14 (3.53 ± 0.70 g vs 11.67 ± 1.68 g, **P < 0.01), and day 21 after CCI surgery (5.26 ± 0.78 g vs 11.13 ± 0.67 g, **P < 0.01). In particular, compared to other time points (days 7 and 21 after CCI surgery), a remarkable decrease in the mechanical threshold occurred on day 14 after CCI surgery (3.53 ± 0.70 g vs 5.48 ± 0.56 g, 5.26 ± 0.78 g, ^##^*P* < 0.01).

The mechanical response threshold of the ipsilateral paw to Von Frey Hair in the following groups: sham surgery (Sham, n = 10) and chronic constriction of the sciatic nerve surgery (CCI, n = 9). Data are means ± SEM. CCI-induced paw withdrawal threshold decrease compared with the sham group, two-way repeated ANOVA, F_1, 51_ = 206.166, *P* < 0.001, post hoc Tukey test: ^**^P < 0.01. CCI-induced paw withdrawal threshold at day 14 compared with the different time points (day 0, 7, 21), post hoc Tukey test: ^##^*P* < 0.01.

### Changes in the thalamic and other areas functional activities on the days0, 7, 14, 21 after CCI surgery

**Fig. 2:**
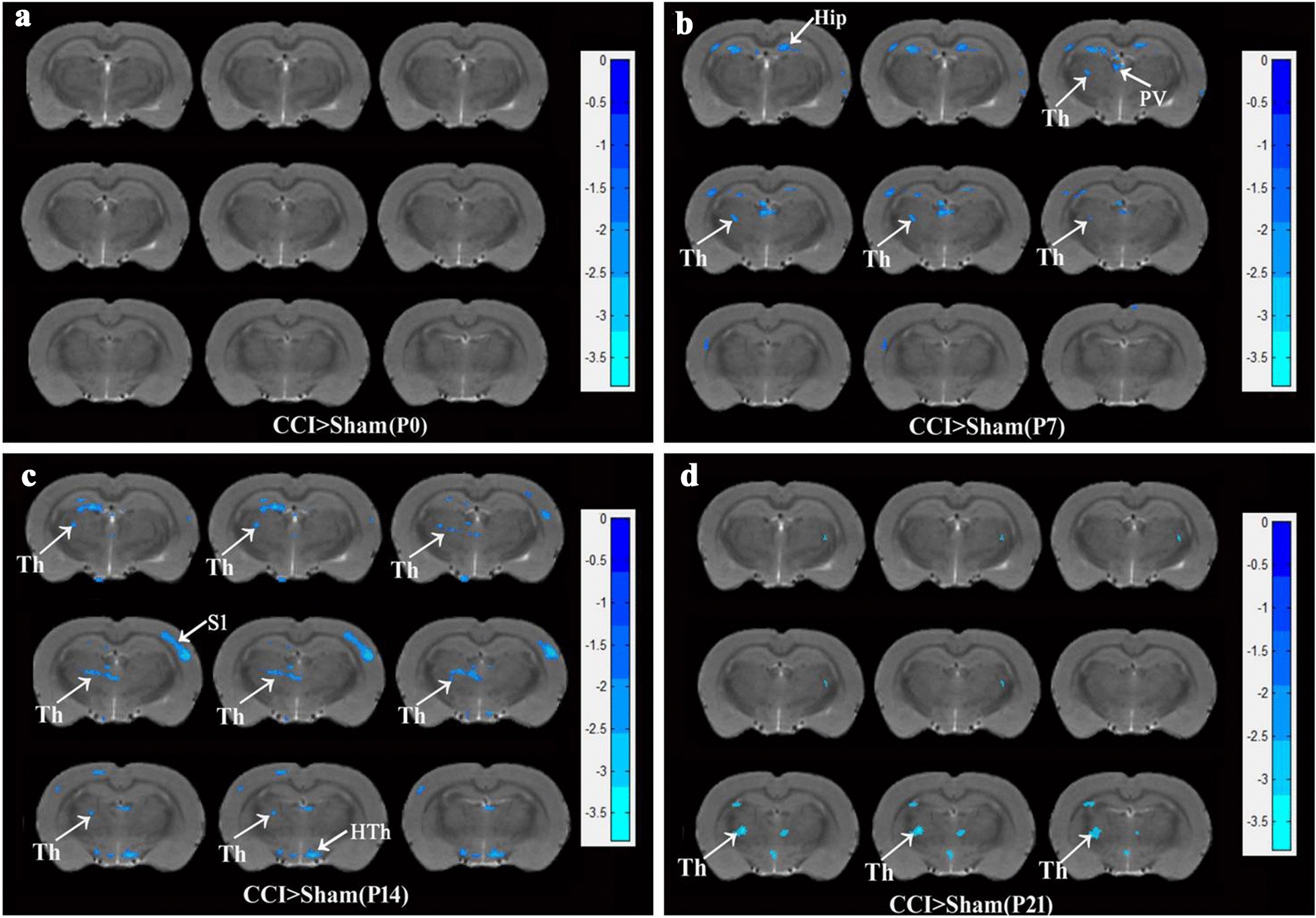
Thalamus and other brain areas ReHo detection in the time course of CCI rats compared with Sham rats. **a** ReHo levels were measured with fMRI on day 0 before surgery between two groups (Sham, n = 10; CCI, n = 9). **b** ReHo on day 7 after surgery. **c** ReHo on day 14 after surgery. **d** ReHo on day 21 after surgery. The coronal section position of thalamus was the range from Interaural 6.36 mm, Bregma − 2.52 mm to Interaural 5.88 mm Bregma − 3.12. CCI-induced Reho decrease compared with the sham group, two-way repeated ANOVA, F_1, 51_ = 221.08, *P* < 0.001, post hoc Tukey test: *P* < 0.01

ReHo was applied to assess resting-state brain activity during the pain state. There were no statistic differences of ReHo between two groups on 0 days without surgery (Fig. 2a). As shown in Fig. 2b, compared to the sham group, decreased ReHo on the left side of the thalamus was found through MRI scanning on day seven after CCI surgery (*P* < 0.01). In addition to the thalamus, decreased ReHo in the bilateral hippocampus and left paraventricular nucleus (PV) of the thalamus were also observed on day 7 after CCI surgery. As shown in Fig. 2c, compared to the sham surgery condition, decreased ReHo on the left thalamus and the right primary sensory cortex (S1) and Hypothalamus (Hth) was found on day 14 after CCI surgery (*P* < 0.01). As shown in Fig. 2d, compared to sham surgery rats, decreased ReHo was found mainly on the left side of the thalamus on day 21 after CCI surgery (*P* < 0.01).

### The alteration of NAA, Glu in the thalamus of CCI rats

**Fig. 3:**
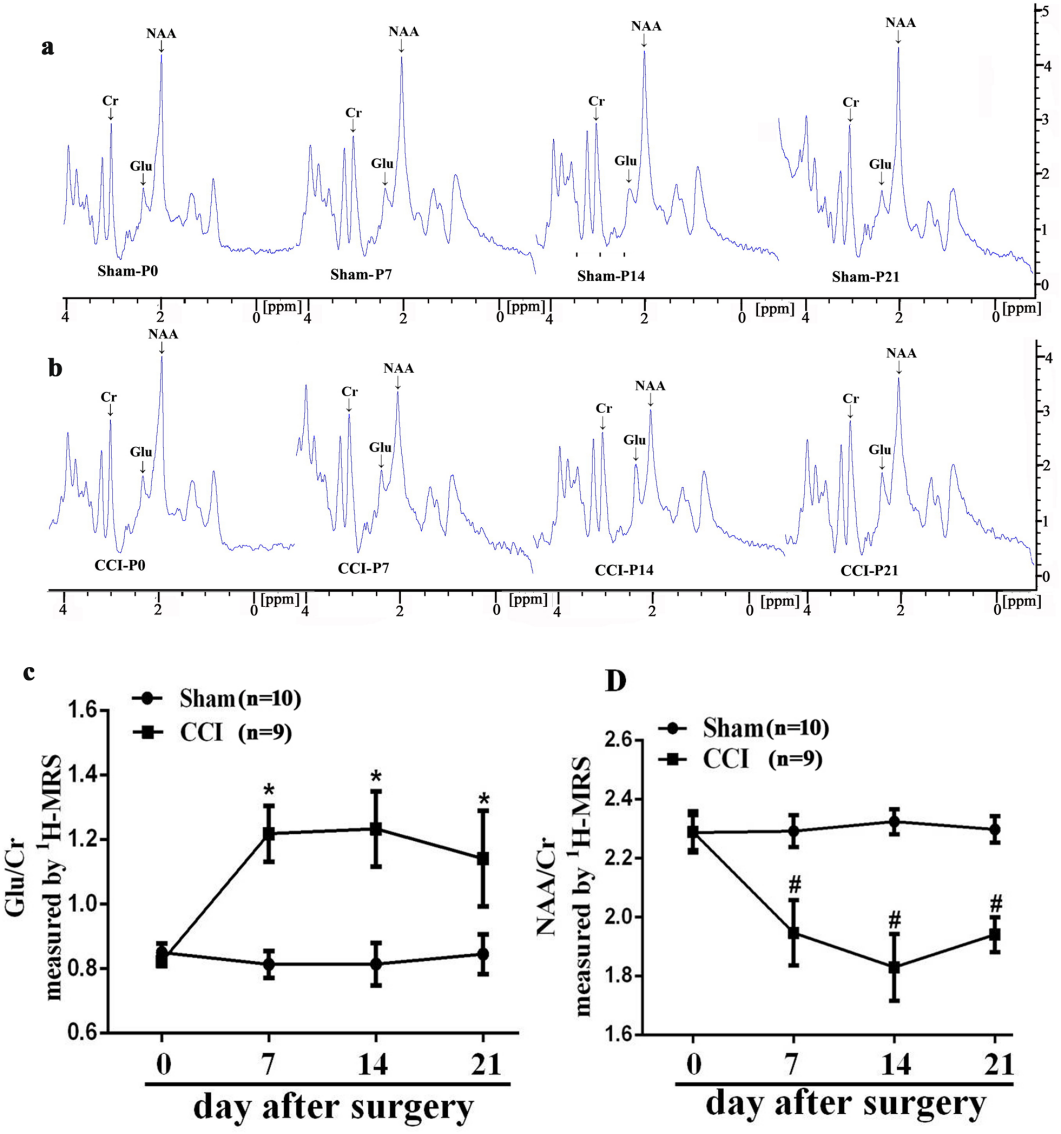

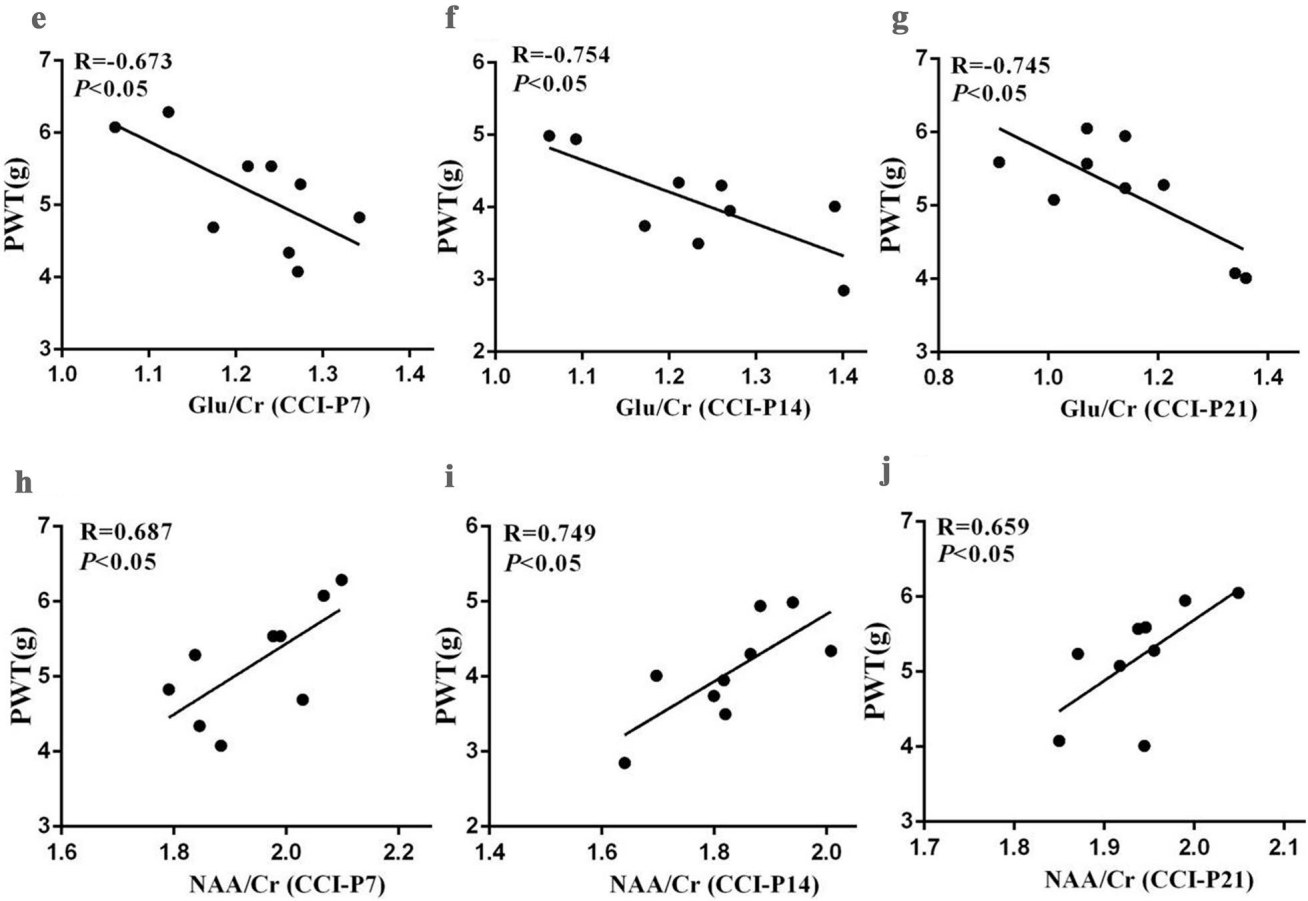
The left thalamus (3 × 3 × 3 mm) ^1^H-MRS detection and correlation analysis between Neurotransmitters and Paw withdrawal thresholds (PWT) in pain condition. **a**–**d** The Glu, NAA in the thalamus were evaluated by MRS scanning at different time points ( baseline, days 7, 14, 21 after surgery) in Sham and CCI rats. **e**–**j** Correlation analysis between thalamic Glu, NAA and mechanical pain thresholds on the days 7, 14, 21 after CCI surgery (CCI, n = 9). CCI-induced Glu increased compared with the sham group, two-way repeated ANOVA, F_1, 51_ = 262.684, *P* < 0.001, post hoc Turkey test: *P < 0.001; CCI-induced NAA decreased compared with the sham group, two-way repeated ANOVA, F_1, 51_ = 250.05, *P* < 0.001, post hoc Turkey test: ^#^*P* < 0.001

To investigate thalamic neurometabolic alterations, MRS was applied following fMRI scanning to evaluate Glu and NAA levels in the thalamus in all experimental animals from day 0 to 21 after CCI surgery. According to the results in Fig. 3a–d, the Glu/Cr ratio in the thalamus was significantly increased compared to the sham group on day 7, 14, and 21 after CCI surgery (*P* < 0.001). However, the NAA/Cr ratio decreased on days 7, 14, and 21 after CCI surgery (*P* < 0.001). There was no statistic difference between the two groups on day 0 after CCI surgery.

As illustrated in Fig. 3e–j, increased concentrations of Glu and decreased levels of NAA in the thalamus were significantly correlated with mechanical allodynia on days 7, 14, 21 after CCI surgery (*P* < 0.05). However, there were no statistical differences between the neurotransmitters and PWT by correlation analysis in sham groups on days 7, 14, 21 after CCI surgery (data not shown). Thus, these results indicate that abnormal release of thalamic Glu and NAA may be one of the causes of neuropathic pain.

### The thalamic functional connectivity varied in the CCI rats

**Fig. 4:**
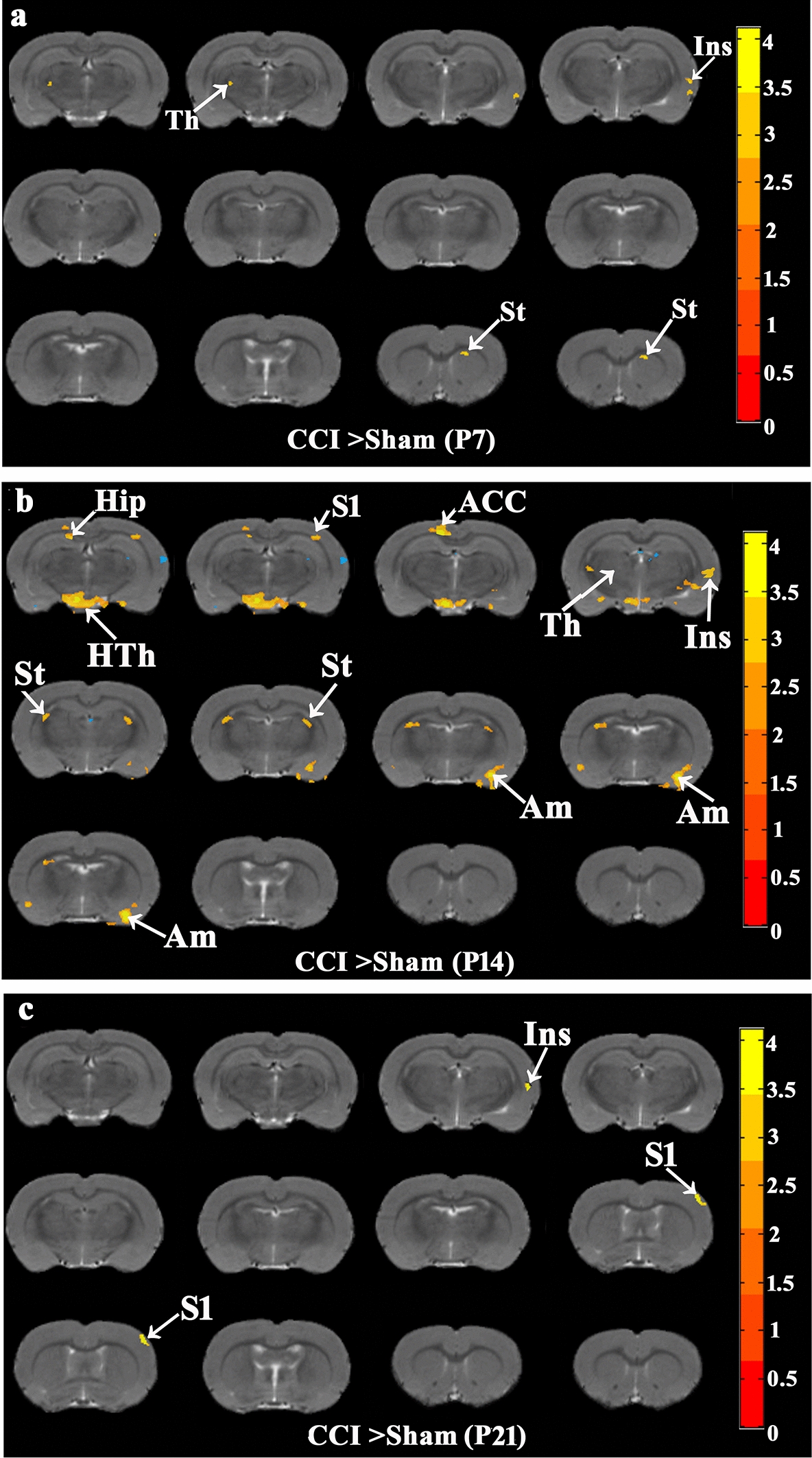
The functional connectivity between the thalamus and other brain regions. **a** Positive brain regions of functional connection on day 7 after CCI surgery. **b** Positive brain regions of functional connection on day 14 after CCI surgery. **c** Positive brain regions of functional connection on day 21 after CCI surgery. Brain coronal section position in Fig. **a**–**c**, first horizontal row: Bregma (− 5.04, − 4.44, − 4.20, − 2.92); second horizontal row: Bregma (− 3.72, − 3.48, − 2.16, − 1.92); third horizontal row: Bregma (− 1.56, − 0.72, 0.24, 0.36)

The functional connectivity between the thalamus and other brain regions is shown in Fig. 4 and Table 1. In Fig. 4a, compared with the sham group, significantly increased functional connectivity was observed between the thalamus and the insula and striatum 7 days after CCI surgery. In Fig. 4b, compared with the sham group, significantly increased functional connectivity was observed between the thalamus and the hypothalamus, amygdaloid body, anterior cingulate cortex, hippocampus, S1, insula and striatum at 14 days after CCI surgery time point. However, we also saw less functional connectivity in the perirhinal cortex. In Fig. 4c, compared with the sham group, significantly increased functional connectivity was observed between the thalamus and the insula and S1 at 21 days after CCI surgery time point. According to Fig. 4 and Table 1, only functional connectivity with the insula and S1 were increased at different time points after CCI surgery.

**Table 1:** Functional connectivity between the thalamus and other brain regions 7, 14, 21 days after CCI surgery Via voxel-sise analysis (*P* < 0.001)

	CCI>Sham		
	Brain regions	Clusters	MAX_T
7 days after surgery	Insular cortex right	10	3.9403
	dorsal thalamus lateral nucleus left	22	3.5523
	Striatum right	18	3.9714
	Amygdaloid body right	223	5.3559
	Amygdaloid body left	9	3.6166
	Hypothalamus tuberal region right	138	4.8916
	Hippocampus left	11	3.6782
14 days after surgery	Hypothalamus mammillary region right	12	3.8328
	Sensory cortex right	30	3.5268
	Striatum left	72	4.0082
	Striatum right	45	3.9295
	Insular cortex right	15	3.437
	Anterior cingulate cortex left	35	4.0408
21 days after surgery	Sensory cortex left	70	4.1288
	Insular cortex right	11	3.5156

## Discussion

In our research, mechanical pain behaviour was observed in the development of neuropathic pain. Previous studies have shown that chronic pain can lead to over-excitation of neurons in different brain regions. These brain areas included spinal cord, thalamus, amygdala, anterior cingulate cortex, and others [15–18]. The thalamus is a gateway for relaying sensory signals to other cortical areas, such as the primary somatosensory cortex and secondary somatosensory cortex [19]. Our results showed the increasing of glutamate and the decrease of NAA levels in the pain conditions induced by CCI. Besides, the dynamic change of glutamate and NAA in the thalamus were significantly correlated with mechanical allodynia at 7, 14, 21 days after CCI surgery. These results indicated that mechanical pain might be related to the increased glutamate release and NAA decrease.

Glutamate is the major excitatory neurotransmitter that modulates neuronal excitability and synaptic transmission in the central nervous system. Some studies have demonstrated that enhanced cortical glutamate levels may contribute to central sensitization during neuropathic pain [20, 21]. Previous MRS studies have also shown that enhanced glutamate levels in the hippocampus might induce hippocampal hyper-excitability during pain conditions [22]. Glutamate levels were significantly increased in the thalamic ventral posterolateral nucleus and ACC in neuropathic pain rats [23, 24]. Clinical research using MRS also showed a significant absolute glutamate increase in the insular cortex, cingulate cortex and thalamus during several pain conditions [25–28]. In our MRS results, increased glutamate releasing in the thalamus during the CCI-induced neuropathic pain, which was caused by excitatory neurons.

NAA is highly concentrated in brain neurons, and its concentration is proportional to the signal conductivity of the neurons. Thus, it has been recognized as a putative marker of neural functionality [29]. It has been found that the higher pain intensity in several neuropathic pain diseases, such as spinal cord injury, trigeminal neuropathy, and painful diabetic neuropathy, is correlated with the lower NAA concentration in the thalamus, which would affect the therapies and prognosis of neuropathic pain. Studies have shown that dysfunction in inhibitory neurons caused by decreased NAA concentrations in neuropathic pain conditions is mediated by the activation of thalamic glial cells [10, 30–35]. In addition to neuropathic pain, the thalamic NAA/Cr ratio detected by MRS was significantly lower in patients with non-neuropathic pain, such as migraine, unilateral pain due to lumbar spine diseases, and osteoarthritis [36–38].

The pain matrix consists of brain regions such as the thalamus, ACC, somatosensory cortex, and insula. It involves the functional reorganization of brain regions sensitive to mechanical allodynia in patients with chronic neuropathic syndromes using multiple neuroimaging methods [39–43]. The matrix is classified into two subsystems according to the different projection pathways of the brain regions: the projections of the lateral thalamic nucleus to the somatosensory cortex mainly constitutes the lateral perceptive system, while the projections between the medial thalamic nucleus group and the ACC constitutes the medial nociceptive system. Thus, it has been concluded that both somatosensory cortex and insula receive sensory inputs from the thalamus, which is the core relay system for injury transmission and is essential for the noxious sensation of peripheral neuralgia. It has been confirmed that thalamic functional abnormalities and structural disruptions that occur in pathological neuropathic pain may be interpreted as spontaneous factors of neuropathic pain [44]. In our fMRI research, significantly increased functional connectivity was observed between the thalamus and other pain-related brain regions, which is similar to previous results revealing that the insula, basal ganglion, amygdala, and limbic system are involved in neuropathic pain at different pathological stages [45]. In addition, in SNI surgical rats, more activations were observed in areas involved in pain modulation, such as the ventral posterolateral nucleus of the thalamus, somatosensory cortex, while deactivations were found in the periaqueductal gray and insula [23].

Our novel finding was that more functional connectivity enhancement was observed between the hypothalamus, amygdala and thalamus at the early post-injury time point in CCI rats, when neuralgia sensitivity was at its peak. Some studies have found that the noxious stimulation of the sciatic nerve in rats might induce medial thalamus and hypothalamus activation during fMRI [16].

The hypothalamus plays a crucial role in the hypothalamic–pituitary–adrenal axis and is impaired under pain conditions. It is worth noting that synaptic plasticity consolidation in the amygdala during the chronic stage of pathological neuralgia may contribute to pain perception and negative emotions [46–48].

Another interesting finding was that the connection between the insular cortex and thalamus was involved in the pathological progression of CCI-induced chronic neuralgia during early and late post-injury time points. It has been confirmed that the insula is the nerve network hub of pain signal transmission. A clinical study has also suggested that chronic neuralgia is associated with fluctuations in the functional network and the activation of excitatory neurons in the insula [49, 50]. As we knew, the dorsal posterior insula is one region that is consistently active at a high level during pain, as seen during functional brain imaging. In addition, trigeminal neuralgia patients exhibit increased functional connectivity between the insula and thalamus [51, 52]. All above these findings have suggested that chronic neuropathic pain produced remarkable and long-lasting functional connectivity enhancement between the thalamus and hypothalamus, amygdale and insula, which might be beneficial for finding the targets of pain drug development in the future.

## Conclusion

At the occurrence and development of neuropathic pain induced by CCI injury, the dynamic changes in thalamic neurotransmitter (glutamate, NAA) concentrations and ReHo contribute to ongoing brain central sensitization and mechanical allodynia. Enhanced thalamus–insular functional connection plays a crucial role in the development of neuropathic pain in CCI rats.

## Data Availability

Not applicable.

## Abbreviations

Am: Amygdaloid body
ACC: Anterior cingulate cortex
CCI: Chronic constriction injury
Cr: Creatine
Glu: Glutamate
Hip: Hippocampus
HTh: Hypothalamus
Ins: Insular cortex
MRI: Magnetic resonance image
MRS: Magnetic resonance spectrum
EPI: Echo-planar imaging
fMRI: Functional magnetic resonance image,
NAA: N-acetylaspartate
PV: Paraventricular nucleus of thalamus
PWT: Paw withdrawal threshold
ReHo: Regional homogeneity
St: Striatum
Th: Thalamus
TE: Echo time
TR: Repetition time

## References

[1] Baron R (2006). Mechanisms of Disease: neuropathic pain-a clinical perspective. Nature Clin Pract Neurol.

[2] Vardeh D, Mannion RJ, Woolf CJ (2016). Toward a mechanism-based approach to pain diagnosis. J Pain.

[3] Friebel U, Eickhoff SB, Lotze M (2011). Coordinate-based meta-analysis of experimentally induced and chronic persistent neuropathic pain. Neuroimage.

[4] Vaculín S, Franek M, Rokyta R (2000). Dorsal rhizotomy changes the spontaneous neuronal activity of nuclei in the medial thalamus. Physiol Res.

[5] Hsueh-Chieh Lu,  Jen-Chuen H,  Ching-Liang Lu (2010). Neuronal correlates in the modulation of placebo analgesia in experimentally-induced esophageal pain: a 3T-fMRI study. Pain.

[6] Whitt JL, Masri R, Pulimood SN (2013). Pathological activity in mediodorsal thalamus of rats with spinal cord injury pain. J Neurosci.

[7] Zhang S, Chiang CY, Xie YF (2006). Central sensitization in thalamic nociceptive neurons induced by mustard oil application to rat molar tooth pulp. Neuroscience.

[8] Zhao X, Xu M, Jorgenson K (2017). Neurochemical changes in patients with chronic low back pain detected by proton magnetic resonance spectroscopy: a systematic review. Neuroimage Clin.

[9] Henderson LA, Peck CC, Petersen ET (2013). Chronic pain: lost inhibition?. J Neurosci.

[10] Sorensen L, Siddall PJ, Trenell MI (2008). Differences in metabolites in pain-processing brain regions in patients with diabetes and painful neuropathy. Diabetes Care.

[11] Grachev ID, Ramachandran TS, Thomas PS (2003). Association between dorsolateral prefrontal N-acetyl aspartate and depression in chronic back pain: an in vivo proton magnetic resonance spectroscopy study. J Neural Transmission.

[12] Amirmohseni S, Segelcke D, Reichl S (2016). Characterization of incisional and inflammatory pain in rats using functional tools of MRI. Neuroimage.

[13] Harris RE, Clauw DJ (2012). Imaging central neurochemical alterations in chronic pain with proton magnetic resonance spectroscopy. Neurosci Lett.

[14] Liang S, Lin Y, Lin B (2017). Resting-state functional magnetic resonance imaging analysis of brain functional activity in rats with ischemic stroke treated by electro-acupuncture. J Stroke Cerebrovasc Dis.

[15] Pere B-V, Judit H, Francisco R (2017). Neuroplasticity of supraspinal structures associated with pathological pain. Anat Rec (Hoboken).

[16] Chao TH, Chen JH, Yen CT (2018). Plasticity changes in forebrain activity and functional connectivity during neuropathic pain development in rats with sciatic spared nerve injury. Mol Brain.

[17] Bill McCarberg, John P (2019). Pain Pathways and Nervous System Plasticity: Learning and Memory in Pain. Pain Med.

[18] Youssef AM, Gustin SM, Nash PG (2014). Differential brain activity in subjects with painful trigeminal neuropathy and painful temporomandibular disorder. Pain.

[19] Chen-Tung Y, Pen-Li L (2013). Thalamus and pain. Acta Anaesthesiol Taiwan.

[20] Kun-Long H, Su-Jane W, Ying-Chou W (2014). Upregulation of presynaptic proteins and protein kinases associated with enhanced glutamate release from axonal terminals (synaptosomes) of the medial prefrontal cortex in rats with neuropathic pain. Pain.

[21] Guida F, Luongo L, Marmo F (2015). Palmitoylethanolamide reduces pain-related behaviors and restores glutamatergic synapses homeostasis in the medial prefrontal cortex of neuropathic mice. Mol Brain.

[22] Ainhoa B, Claudia F-M, Sarah L (2018). Longitudinal structural and functional brain network alterations in a mouse model of neuropathic pain. Neuroscience.

[23] Hubbard CS, Khan SA, Xu S (2015). Behavioral, metabolic and functional brain changes in a rat model of chronic neuropathic pain: a longitudinal MRI study. Neuroimage.

[24] Ghanbari A, Asgari AR, Kaka GR (2014). In vivo microdialysis of glutamate in ventroposterolateral nucleus of thalamus following electrolytic lesion of spinothalamic tract in rats. Exp Brain Res.

[25] Gutzeit A, Meier D, Froehlich JM (2013). Differential NMR spectroscopy reactions of anterior/posterior and right/left insular subdivisions due to acute dental pain. Eur Radiol.

[26] Nicolás F, Eva A, Laura V (2014). Higher glutamate+glutamine and reduction of N-acetylaspartate in posterior cingulate according to age range in patients with cognitive impairment and/or pain. Acad Radiol.

[27] Kun Lv, Wenwen S, Rui T (2018). Neurotransmitter alterations in the anterior cingulate cortex in Crohn's disease patients with abdominal pain: a preliminary MR spectroscopy study. Neuroimage Clin.

[28] Ye-Ha J, Hyeonjin K, Yeon SJ (2018). Peripheral and central metabolites affecting depression, anxiety, suicidal ideation, and anger in complex regional pain syndrome patients using a magnetic resonance spectroscopy: a pilot study. Psychiatry Investig.

[29] Baslow Morris H, Hrabe J, Guilfoyle DN (2007). Dynamic relationship between neurostimulation and N-acetylaspartate metabolism in the human visual cortex: evidence that NAA functions as a molecular water pump during visual stimulation. J Mol Neurosci.

[30] Maheshwari SR, Fatterpekar GM, Castillo M (2000). Proton MR spectroscopy of the brain. Semin Ultrasound CT MRI.

[31] Pattany Pradip M, Yezierski Robert P, Widerström-Noga Eva G (2002). Proton magnetic resonance spectroscopy of the thalamus in patients with chronic neuropathic pain after spinal cord injury. Am J Neuroradiol.

[32] Widerström-Noga E, Cruz-Almeida Y, Felix Elizabeth R (2015). Somatosensory phenotype is associated with thalamic metabolites and pain intensity after spinal cord injury. Pain.

[33] Gustin Sylvia M, Peck Chris C, Wilcox Sophie L (2011). Different pain, different brain: thalamic anatomy in neuropathic and non-neuropathic chronic pain syndromes. J Neurosci.

[34] Sei F, Miyuki M, Toshiro I (2006). N-Acetylaspartate concentrations in the thalami of neuropathic pain patients and healthy comparison subjects measured with (1)H-MRS. Magn Reson Imaging.

[35] Gustin SM, Wrigley PJ, Youssef AM (2014). Thalamic activity and biochemical changes in individuals with neuropathic pain after spinal cord injury. Pain.

[36] Tao Gu, Lei L, Yun J (2018). Acupuncture therapy in treating migraine: results of a magnetic resonance spectroscopy imaging study. J Pain Res.

[37] Shigemura T, Kishida S, Eguchi Y (2012). Proton magnetic resonance spectroscopy of the thalamus in patients with osteoarthritis of the hip. Bone Joint Res.

[38] Shoji Y, Shin-ichi K, Shin-ichi K (2013). Assessment of pain due to lumbar spine diseases using MR spectroscopy: a preliminary report. J Orthop Sci.

[39] Lino B, Susie M, Shelly B (2006). Trigeminal neuropathic pain alters responses in CNS circuits to mechanical (brush) and thermal (cold and heat) stimuli. Neurosci.

[40] Witting N, Kupers RC, Svensson P (2006). A PET activation study of brush-evoked allodynia in patients with nerve injury pain. Pain.

[41] Mouraux A, Diukova A, Lee MC (2011). A multisensory investigation of the functional significance of the“pain matrix”. Neuroimage.

[42] Moseley GL (2003). A pain neuromatrix approach to patients with chronic pain. Man Ther.

[43] Iannetti G, Mouraux A (2010). From the neuromatrix to the pain matrix (and back). Exp Brain Res.

[44] Roland P (2016). Functional brain imaging: what has it brought to our understanding of neuropathic pain? A special focus on allodynic pain mechanisms. Pain.

[45] Okihiro O, Kazuya I, Ryo O (2018). Sequential variation in brain functional magnetic resonance imaging after peripheral nerve injury: A rat study. Neurosci Lett.

[46] Yonghui G, Chen S, Qiuling X (2013). Proteomic analysis of differential proteins related to anti-nociceptive effect of electroacupuncture in the hypothalamus following neuropathic pain in rats. Neurochem Res.

[47] Ikeda R, Takahashi Y, Inoue K (2007). NMDA receptor-independent synaptic plasticity in the central amygdala in the rat model of neuropathic pain. Pain.

[48] Chang C, Shyu BC (2001). A fMRI study of brain activations during non-noxious and noxious electrical stimulation of the sciatic nerve of rats. Brain Res.

[49] Ching Y, Wang C, Tay T (2018). Altered sensory insular connectivity in chronic postsurgical pain patients. Front Hum Neurosci.

[50] Han J, Cha M, Kwon M (2016). In vivo voltage-sensitive dye imaging of the insular cortex in nerve-injured rats. Neurosci Lett.

[51] Peyron R, Fauchon C (2019). Functional imaging of pain. Rev Neurol (Paris).

[52] Wang Y, Zhang Y, Zhang J (2018). Structural and functional abnormalities of the insular cortex in trigeminal neuralgia: a multimodal magnetic resonance imaging analysis. Pain.

